# Olfactory Neuroblastoma: A Rare Cause of External Ophthalmoplegia, Proptosis and Compressive Optic Neuropathy

**DOI:** 10.4274/tjo.81568

**Published:** 2018-04-25

**Authors:** Ömer Kartı, Mehmet Özgür Zengin, Ozan Çelik, Taşkın Tokat, Tuncay Küsbeci

**Affiliations:** 1 University of Health Sciences, İzmir Bozyaka Training and Research Hospital, Ophthalmology Clinic, İzmir, Turkey; 2 University of Health Sciences, İzmir Bozyaka Training and Research Hospital, Otolaryngology Clinic, İzmir, Turkey

**Keywords:** External ophthalmoplegia, compressive optic neuropathy, olfactory neuroblastoma, proptosis

## Abstract

Olfactory neuroblastoma (ONB), which is a neuroectodermal tumor of the nasal cavity, is a rare and locally aggressive malignancy that may invade the orbit via local destruction. In this study, we report a patient with proptosis, external ophthalmoplegia, and compressive optic neuropathy caused by ONB. A detailed clinical examination including ocular imaging and histopathological studies were performed. The 62-year-old female patient presented to our clinic with complaints of proptosis and visual deterioration in the left eye. Her complaints started 2 months prior to admission. Visual acuity in the left eye was counting fingers from 2 meters. There was relative afferent pupillary defect. She had 6 mm of proptosis and limitation of motility. Fundus examination was normal in the right eye, but there was a hyperemic disc, and increased vascular tortuosity and dilation of the retinal veins in the left eye. Computerized tomography and magnetic resonance imaging of the brain and orbits demonstrated a large heterogeneous mass in the left superior nasal cavity with extensions into the ethmoidal sinuses as well as into the left orbit, compressing the medial rectus muscle and optic nerve. Endoscopic biopsy of the lesion was consistent with an ONB (Hyams’ grade III). Orbital invasion may occur in patients with ONB. Therefore, it is important to be aware of this malignancy because some patients present with ophthalmic signs such as external ophthalmoplegia, proptosis, or compressive optic neuropathy.

## Introduction

Olfactory neuroblastoma (ONB), also referred to as esthesioneuroblastoma, was first described by Berger in 1924. It is a rare neuroectodermal malignant tumor of the nasal cavity originating from the olfactory neuroepithelium.^1^ ONB constitutes 2-6% of all malignancies of the nasal cavity and paranasal sinuses. The incidence is highest in the second and sixth decades of life.^2,3,4^ This fast-growing tumor can be asymptomatic until it fills the nasal cavity and causes obstruction and/or epistaxis. It may spread into the cranium, orbit, and paranasal sinuses. Orbital invasion can lead to vision loss, ophthalmoplegia, and proptosis. In this study, we present the clinical features of a 62-year-old female patient with olfactory neuroblastoma showing orbital invasion.

## Case Report

**Figure 1:**
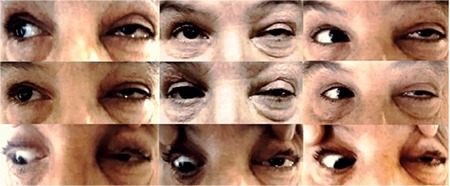
The nine cardinal positions of gaze. Periorbital edema and proptosis are seen in the left eye and movements are restricted in all directions except upgaze

**Figure 2:**
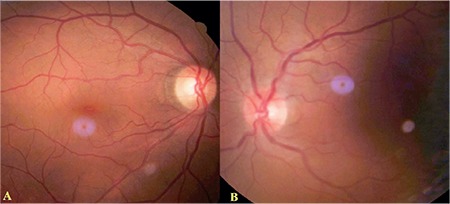
Color fundus photograph: the right eye appears normal (A), while the left eye shows hyperemic optic disc and increased vascular tortuosity and caliber of the retinal veins (B)

**Figure 3:**
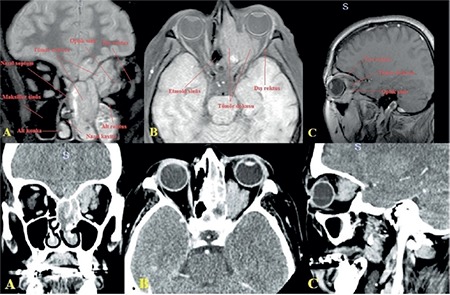
Magnetic resonance and computed tomography images: A mass is seen completely filling the left nasal cavity and anterior/posterior ethmoid sinuses, extending beyond the medial rectus muscle into the cone and displacing the optic nerve inferiorly in coronal (A), transverse (B), and sagittal (C) images

**Figure 4:**
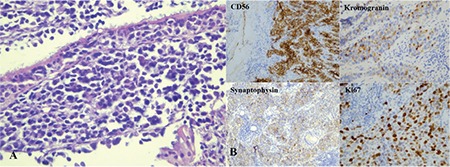
(A) Relatively uniform, noncohesive tumor cells with inconspicuous nucleoli, indistinct nuclear membranes, and low cytoplasmic ratio (hematoxylin and eosin; x400). (B) Immunohistochemical analysis of the tumor cells shows CD56, chromogranin, and synaptophysin positivity and Ki-67 labeling index indicated high proliferation

A 62-year-old female patient presented to our unit with complaints of double vision. She stated that her double vision had started approximately one month earlier. Neuroophthalmologic examination revealed bilateral light responses with no afferent pupil defect. Color vision test with Ishihara (14 card print) was 14/14 in both eyes. The patient exhibited abnormal head posture (face turn to right) and hypertropia of the left eye (10 prism diopters). Slight restriction of left eye movement was noted in down/right gaze. There were no obvious limitations in the other positions of gaze. In the Bielschowsky head tilt test, hypertropia increased when the head was tilted to the left. Best corrected visual acuity on Snellen chart was 10/10 in both eyes. Anterior segment and fundus examinations were normal in both eyes. The patient was diagnosed with left fourth cranial nerve palsy and cranial imaging was requested to establish etiology; however, the patient did not return for follow-up. Approximately 2 months later, our unit was consulted again regarding the patient due to complaints of reduced vision and pain in the left eye. She also had complaints of progressive swelling in the left half of her face and enlargement of her left eye for about one month. On neuroophthalmologic examination, light response was normal in the right eye and weak in the left eye. Relative afferent pupil defect was noted in the left eye. Right eye movements were unrestricted in all directions, while left eye movements were restricted in all directions with minimal upward gaze (Figure 1). Proptosis was noted in the left eye, and Hertel exophthalmometer measurements were 18 mm for the right eye and 24 mm for the left eye. Best corrected visual acuity on Snellen chart was 9/10 in the right eye and counting fingers from 2 m in the left eye. Color vision score was 14/14 in the right and 0/14 in the left eye. Chemosis and upper lid edema were observed in the left eye on slit-lamp examination. The anterior segment appeared normal. Intraocular pressure measured by Goldmann applanation tonometry was 15 mmHg in both eyes. Fundus examination was normal in the right eye, while optic disc hyperemia and increased tortuosity and caliber of the retinal vessels were observed in the left eye (Figure 2). Reliable results could not be obtained in visual field and visual evoked potential tests. Computed tomography scans revealed a mass filling the left nasal cavity and anterior/posterior ethmoid sinuses, extending beyond the medial rectus muscle into the cone and compressing the optic nerve. Orbital magnetic resonance imaging revealed a soft tissue mass with homogeneous enhancement, hypointense in T1-weighted sequences and hyperintense in T2-weighted sequences, which appeared to be displacing the optic nerve inferiorly (Figure 3). Based on the results of histopathological examination of a punch biopsy obtained from the nasal passage, the patient was diagnosed with olfactory neuroblastoma (Hyams grade III). Immunohistochemical staining for differential diagnosis of the round cell tumor was negative for CD2, CD3, CD20, CD38, cytokeratin, CD99, HMB45 and GFAP. A small number of cells were positive for CD56, chromogranin, and synaptophysin, while very few cells were positive for BCL2, NF, and S100. Ki-67 labeling index was 25% (Figure 4).

## Discussion

The most common symptom of ONB was reported by Dulguerov and Calcaterra^5^ as unilateral nasal obstruction, which they observed in 71% of 26 patients. Other common symptoms are anosmia, headache, lacrimation, proptosis, and reduced vision. In rare cases, ONB secretes antidiuretic hormone (ADH) and causes syndrome of inappropriate ADH secretion, or produces ectopic adrenocorticotropic hormone and leads to Cushing’s syndrome.^6^ In a large series including 38 cases of ONB, Rakes et al.^7^ reported that 53% of patients had orbital or ocular symptoms and that the most common symptoms were periorbital pain and lacrimation. In addition, they determined that diplopia was the initial ocular symptoms in 8% of the patients.^7^ Most patients develop diplopia secondary to tumor invasion of the orbit. On the other hand, ONB can cause cranial nerve palsy without orbital invasion. Rakes et al.^7^ reported cranial nerve palsy in a patient without orbital involvement in their series. Lee and Tang^8^ also reported third cranial nerve palsy with pupil involvement due to mass extension to the cavernous sinus, without orbital involvement. Fourth cranial nerve palsy was the first clinical finding in our case; however, as the patient did not attend follow-up, the diagnosis was established after she presented a second time with ophthalmoplegia and proptosis, likely due to expansion of the tumor tissue within the orbit. ONB-associated optic neuropathy may develop due to compressive and/or infiltrative causes.^9^ In our case, we observed that the tumor inferiorly displaced the optic nerve in orbital imaging and thus believed neuropathy was a result of compression. However, it should be noted that tumor infiltration cannot be excluded without histopathological evaluation.

Hyams et al.^10^ divided ONB into four histological grades. The grading system is based on evaluations of mitotic index, necrosis, rosette formation, calcification, pleomorphism, lobular structure, and the neurofibrillary matrix. Grade III and IV have been associated with poor prognosis.^10^ Currently, standard treatment of ONB consists of total surgical excision (open or endoscopic craniofacial resection) and adjuvant radiotherapy.^11,12^ The most common site of metastasis in ONB patients is the cervical lymph nodes, seen in 20-25% of patients. Neck metastases are detected in 5-8% of patients at time of diagnosis. 

In brief, ONB is a rare endonasal tumor. It may manifest with initial findings of cranial nerve palsy, proptosis, and/or compressive optic neuropathy. Therefore, this rare etiology must be considered in the differential diagnosis of cranial nerve palsy, external ophthalmoplegia, and proptosis.

## References

[1] Diaz EM Jr, Johnigan RH, Pero C, El-Naggar AK, Roberts DB, Barker JL, DeMonte F (2005). Olfactory neuroblastoma: The 22-year experience at one comprehensive cancer center. Head Neck..

[2] Bradley PJ, Jones NS, Robertson I (2003). Diagnosis and management of esthesioneuroblastoma. Curr Opin Otolaryngol Head Neck Surg..

[3] Lund VJ, Howard D, Wei W, Spittle M (2003). Olfactory neuroblastoma: past, present, and future?. Laryngoscope.

[4] Song CM, Won TB, Lee CH, Kim DY, Rhee CS (2012). Treatment modalities and outcomes of olfactory neuroblastoma. Laryngoscope..

[5] Dulguerov P, Calcaterra T (1992). Esthesioneuroblastoma: the UCLA experience 1970-1990. Laryngoscope..

[6] Wenig BM (2016). Atlas of Head and Neck Pathology. 3rd ed.

[7] Rakes SM, Yeatts RP, Campbell RJ (1985). Ophthalmic manifestations of esthesioneuroblastoma. Ophthalmology..

[8] Lee AG, Tang RA (2000). Third nerve palsy as the presenting manifestation of esthesioneuroblastoma. J Neuroophthalmol..

[9] Chew FLM, Nurliza K, Prepageran N, Mun KS, Waran V (2009). An Unusual Orbital Presentation of Olfactory Neuroblastoma. Neuro-Ophthalmology..

[10] Hyams VJ, Batsakis JG, Michaels L (1988). Tumors of the Upper Respiratory Tract and Ear, Atlas of Tumor Pathology. Washington DC: Armed Forces Institute Press; Olfactory neuroblastoma.

[11] Howell MC, Branstetter BF 4 th, Snyderman CH (2011). Patterns of regional spread for esthesioneuroblastoma. AJNR Am J Neuroradiol..

[12] Zanation AM, Ferlito A, Rinaldo A, Gore MR, Lund VJ, McKinney KA, Suárez C, Takes RP, Devaiah AK (2010). When, how and why to treat the neck in patients with esthesioneuroblastoma: A review. Eur Arch Otorhinolaryngol..

